# Platelet Membrane Transporters in Health and Disease: Basic Biology and Clinical Relevance

**DOI:** 10.3390/biom16101537

**Published:** 2026-10-21

**Authors:** Deepa Gautam, Ana Kasirer-Friede

**Affiliations:** 1 Brigham and Women’s Hospital, Harvard Medical School, Boston, MA 02115, USA; 2 Department of Medicine, University of California, La Jolla, CA 92037, USA

**Keywords:** platelet, metabolism, membrane transporters, megakaryocytes

## Abstract

Platelet surface adhesion receptors and intracellular signaling proteins have been the focus of platelet research for decades, with the aim of understanding how they contribute to hemostatic and thrombotic functions. However, membrane transporters and their respective roles in platelets and megakaryocytes have received relatively little attention, despite their importance for cellular regulation. Platelets harbor members of two principal structurally dissimilar large families of transporters differing in their energy source: ABC (ATP-binding cassette) transporter family and the solute carrier (SLC) family. Family members comprise integral membrane proteins that control the uptake, distribution and excretion of a variety of nutrients and metabolites, lipids, iron and drugs across concentration gradients, thereby exerting significant influence over cellular metabolism and platelet activation. In addition to transporters found in the plasma membrane, platelet transporters may also localize to lysosomal, mitochondrial and secretion granule membranes or to additional organelles. Interestingly, the impact of platelet transporters does not appear to be limited to canonical platelet functions but may extend to recently established platelet interactions with immune cells and selected tissues, with repercussions on health and disease. More recently, members of these two families have been recognized as modulators of drug efficacy and proposed as therapeutic targets. In this review we describe transporter structure, corresponding molecular mechanisms of cargo transport, functional significance, and transporter regulation, within a platelet framework.

## 1. Introduction

Platelets, though anucleate, play roles in a wide range of disease processes, in particular, hemostasis and thrombosis, and support other necessary functions for diverse cells and organ systems throughout the body [1]. Their intracellular storage granules are replete with myriad compounds that aid these functions, but platelets contain subcellular organelles such as mitochondria, a modified endoplasmic reticulum referred to as the dense tubular system, and remnants of a Golgi network [2,3]. One of the earliest events to occur upon activation is the disk to sphere transformation, referred to as shape change, which is accompanied by loss of the microtubular circumferential band. Structurally, platelets are endowed with a complex cytoskeletal network that is necessary for spreading, membrane pseudopod formation and adhesion stabilization and also harbor an intricate network of intracellular signaling molecules and second messengers. The platelet surface is highly populated with receptors for adhesion molecules, channels and membrane transporters [4].

In this review, we will focus on two large families of platelet membrane transporters, the ATP-binding cassette (ABC) and solute carrier (SLC) families. Mutations or deletions of platelet membrane transporters are causative for several diseases and may contribute to drug resistance. Although proteomic and genomic data have indicated a wide array of platelet expressed ABCs and SLCs [5,6] not all have been confirmed experimentally; thus, in this article we will focus on those with existing associated data (Table 1).

## 2. Platelet ABC and SLC Membrane Transporter Structures and Regulation Structure

Structural data for membrane transporters was originally mostly derived from X-ray crystallography structures of homologous bacterial proteins [59,60] and used to generate in silico human protein structures that could later be verified using mouse proteins. More recently, Cryo-EM has allowed for resolution of structures at close to atomic level resolution, that can be extrapolated to other transporters by molecular modeling approaches, and then applied to simulate protein–protein interactions with substrates and dynamic behavior as the proteins move through their transporter cycle [61,62,63].

**Figure 1:**
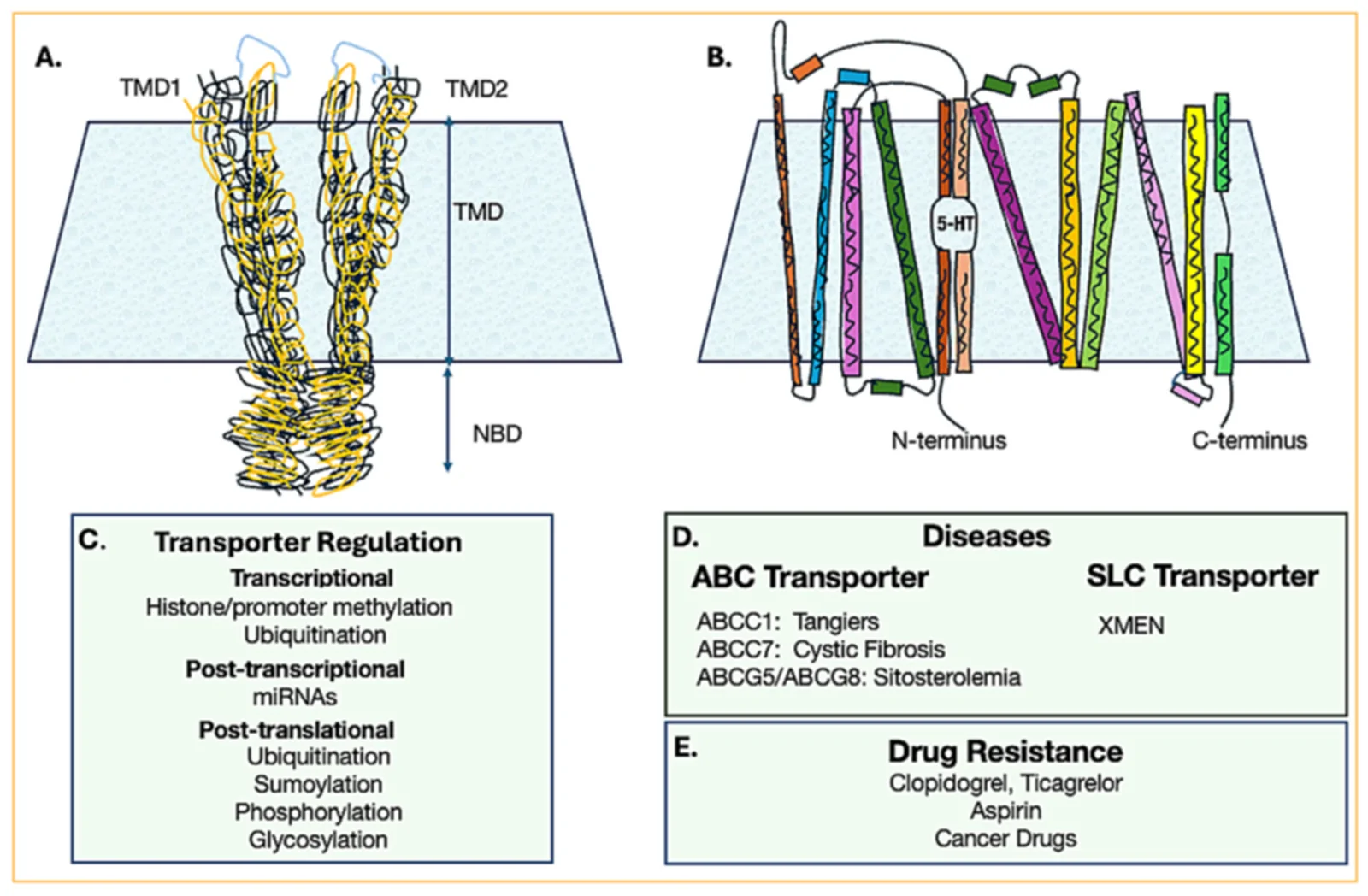
Transporter structure, regulation and disease impact. ABC family membrane transporters share more conserved structures than SLC transporter family members. (**A**) Representative ABC transporter showing nucleotide binding domains and the regulatory domain. (**B**) Schematic of *SLC6a4*, serotonin transporter (SERT) showing multiple transmembrane domains and serotonin (5-HT) binding pocket. (**C**) Modes of transporter regulation. (**D**) Disease affected by genetic mutation or deletion of transporters. (**E**) Drug efficacy affected by membrane transporter activity.

Human ABCs comprise seven subfamilies, with A, B, C, and G playing key roles in transport and are definitively documented in platelets (Table 1). ABC transporters hydrolyze ATP to transfer diverse classes of small molecules against their electrochemical gradient across cell membranes, mostly serving to excrete cargo from the cell. ABC transporters share similar structural scaffolds: there are two clusters consisting of six transmembrane helices that constitute substrate binding/recognition sites, along with two nucleotide binding domains (NBDs), arranged in a head-to-tail configuration that together form two separate nucleotide binding sites (NBS), and a central regulatory (R) domain (Figure 1A). The transmembrane domains (TMDs) are the site of substrate recognition and translocation. To effect substrate export coupled to ATP hydrolysis, transporters cycle through binding of a substrate to the TMDs, binding of two ATP/Mg molecules to the NBS, NBD dimerization, and large conformational changes as the transporter switches between high-affinity inward to low-affinity outward-facing conformers. This is followed by ATP hydrolysis, and phosphate, ADP and substrate release concomitant with NBD dissociation in the low-affinity outward-facing position to reset the transporter to the ground state for the next cycle [17,64,65,66]. Alternate models for transport have also been proposed (as discussed in some references provided). Molecular modeling has furthermore identified flexible linkers that may regulate transporter function by providing sites for post-translational modifications (PTM) [67,68]. Variations in ABC structures and efflux mechanisms may exist. As an example, the ABCC proteins harbor an N-terminal extension that may include an additional TM domain, and/or variable linkers [17]. Even more distinct is the CFTR transporter, ABCC7 whose NBS is degenerate, thus greatly limiting hydrolysis activity. Instead, it functions as an ATP-gated ion channel for chloride and bicarbonate ions. A large regulatory (R) domain, unique to CFTR, connects the two halves of the protein and plays an essential role in regulating the channel activity [69]. Notably, several ABC transporters, by virtue of their efflux functions, also play important roles in drug resistance (see Section 6).

SLCs are the largest class of membrane transporters, comprising close to 500 members, organized into approximately 80 SLC families, and exhibiting different structural classes or alpha folds. They are important for the import of a wide range of nutrients and metabolites as well as drugs. Key differences that distinguish the SLC superfamily of transporters from the ABC family are their lack of direct energy generation through ATP hydrolysis and their ability to import substrates across membranes. SLCs may operate by facilitated diffusion (passive transport), whereby a single substrate is moved along its concentration gradient in an energy independent fashion or by secondary active transport, with energy coupling provided by the unidirectional movement of one type of ion coupled to the movement of another type of ion or solute (symport), or by counter transport (antiport), down their electrochemical gradients.

SLC structures are highly diverse, often characterized by a cluster of 7–14 hydrophobic membrane-spanning segments (representative modeled structure of serotonin transporter, Figure 1B), which when bundled allow passageway for substrates through the membrane. Interestingly, human SLC transporters belonging to structurally dissimilar families or folds can have chemically comparable substrates. Three types of alternating access models have been proposed for SLC transporter mechanisms: (a) the Rocker-switch, where the amino and carboxy terminal halves may oscillate between an outward-facing state and an inward-facing state along the symmetry axis perpendicular to the membrane; (b) the Gated-Pore, where the substrate binding site is bounded by two gates, an outward-facing one which can open to allow binding of the substrate, followed by opening of the second inward-facing gate to allow release of the substrate into the cytosol; and (c) the Elevator, which involves elevating the transport domain within the membrane to the extracellular site, opening to allow substrate binding, sliding downwards across the membrane and finally opening to release the substrate. There is evidence for all three mechanisms; however, they may be differentially implemented by the various SLC transporters [70,71,72,73,74,75].

**Regulation.** Transporter transcription, occurring primarily in megakaryocytes since platelets are anucleate, depends on transcription factor binding to accessible promoter regions in DNA and may be modulated by enhancers or silencers, the cellular environment, the presence of co-regulators, e.g., c-JUN, c-FOS, and ATF2, or reactive oxygen species, e.g., by heat shock factor 1 and 27 and NF-kB, for *ABCB1*/MDR1, a representative transporter protein [76]. However, transcription factors RUNX1, GATA1, STAT3, NF-κB and PPAR have been identified in platelets and hypothesized to have non-transcriptional activities [77]. Residual megakaryocyte-derived mRNA in platelets allows de novo synthesis of proteins for rapid response to vascular injury or inflammation at the time of activation [78]. miRNAs, and histone acetylation also have been noted to modulate transporter expression [10,76], and are areas of interest for understanding critical nodes in transporter regulation.

Transporters have dynamic structures despite their lipid embedding and high hydrophobicity. Addition of post-translational modifications (PTMs) may largely depend on exposed amino acid sequence, physical, chemical and structural constraints on the surface, cellular co-expression of modifier enzymes, and various adapter proteins primed or re-localized through signaling cascades, to ensure execution of the PTM [79]. Most modifications are cytoplasmic and must be solvent accessible. Common post-translational covalent additions include phosphorylation (reversible via phosphatases), ubiquitination (mono- or poly- most commonly added to lysine chains), glycosylation, and sumoylation. Typically, solvent-accessible modifications are added to protein tails or flexible linker regions but may require dynamic molecular movement to expose sites, e.g., in juxta transmembrane regions that may otherwise be buried in the lipid [79]. Actual modifications added in the platelet environment would require appropriate expression levels of modifier proteins and favorable affinities for the substrate compared to those of competing binding partners.

## 3. Megakaryopoiesis/Thrombopoiesis

Platelets are generated through a specialized developmental program that begins with hematopoietic stem and progenitor cells in the bone marrow and ends with the release of functional platelet fragments into the circulation [80]. While differing hypotheses exist regarding whether blood cell development proceeds through a hierarchy of distinct precursor lineages, or by clonal expansion of pre-lineage/HSC-proximal cells, genomic and protein studies can differentially identify cells at various stages, including hematopoietic stem cells, myeloid erythroid progenitors, common myeloid progenitors and megakaryocytes, the immediate precursors to platelets [81,82]. During maturation, megakaryocytes undergo endomitosis, a specialized process in which DNA replication continues without complete cell division. This produces large polyploid cells with abundant cytoplasm. The demarcation membrane system (DMS) divides the cytoplasm into small platelet territories and contributes membrane for proplatelet formation. Rather than producing platelets by simple cytoplasmic breakdown, mature megakaryocytes undergo an organized remodeling process in which they extend long, branching proplatelet projections through the bone marrow sinusoids. Microtubule-based cytoskeletal rearrangement helps elongate proplatelets and deliver subcellular proteins and megakaryocyte-derived RNAs to developing platelet buds [83] that serve as platforms for the deposit of granules and organelles [84]. As a result, newly formed platelets enter the blood as small anucleate cells that are already equipped with receptors, α-granules, dense granules, mitochondria, and the signaling machinery required for rapid responses to vascular injury [84,85,86,87]. The binding of the cytokine, thrombopoietin (TPO) to its receptor, MPL on platelets and megakaryocytes regulates platelet production in a feedback-dependent manner [88].

The expression of relevant proteins at the appropriate levels and combinations for each developmental stage, is essential for normal thrombopoiesis. Defects in production of platelets may be due to individual protein deficiencies, inefficient priming of megakaryocyte and platelet production in the bone marrow (BM) niche, and abnormal differentiation and fragmentation of megakaryocytes to platelets [5]. While TPO and MPL receptors, NFE-2 and other key transcription factors are among the well-known direct mediators of platelet production, little focus has been placed on the contribution of membrane transporters in this process.

When considering the role of membrane transporters, it is important to keep in mind that platelet numbers in plasma are a consequence of both platelet production and clearance, thus some caution is advised when considering the etiology of thrombocytopenia, where platelets may be either of normal or enlarged (macrothrombocytopenia) size. In this section, we will focus on roles for transmembrane transporter family members in megakaryocyte and platelet generation, as well as in platelet stability. Members of both select ABC and SLC transporters may be involved in regulating megakaryocytic progenitor, megakaryocyte and platelet numbers and their resting morphology and size. Several ABC transporters were identified by combining a TaqMan low-density array RNA screening platform with a liquid chromatography/mass spectrometry method to visualize RNA and membrane proteins in CD34 positive TPO-treated cells, megakaryocytes and megakaryocyte–erythroid progenitors [89]. Whereas transcripts suggested about 25 genes with differential expression with respect to progenitor cells, of these five were identified by mass spectrometry and localized by immunofluorescence: *ABCC4*, *ABCA7*, *ABCB4*, *ABCC1*, and *ABCC3*. The expression of ABCC1, ABCC3, ABCB4 and ABCA7 were also confirmed by Western blot and immunofluorescence [9], and additional SLC transporters were identified by genomics studies [43].

***ABCC4/*MRP4**. The ABCC4 transporter, also known as multidrug resistance-associated protein 4 (MRP4), is one of the first transporters to be identified in platelets and is the highest membrane transporter expressed [7]. ABCC4 transports cyclic nucleotides such as cAMP and cGMP, as well as some lipid mediators, e.g., TxB2, and eicosanoids. Expression of the ABCC4 transporter increases as differentiation progresses from CD34+ cells to yield 465% higher expression in platelets than in progenitor cells [7]. A meta-analysis of GWAS studies, indicated that *ABCC4* was one of 68 genomic loci reliably associated with platelet count and volume [14]. Investigation of ABCC4 as a modulator of thrombopoiesis using *Abcc4*^−/−^ fetal liver cultures, modified human MEG-01 cells, or the delayed platelet recovery following a 5-FU flush, all posited a role for ABCC4 in thrombopoiesis, since there was decreased proplatelet formation, and megakaryocyte polyploidization in *Abcc4*^−/−^ mice [89]. According to analyses of public data ranking the genes by their total contact number with their promoter, *ABCC4* ranked higher than genes with known established roles in megakaryopoiesis (*GATA1*, *FLI1*, *TAL1* and *MEIS1*) when examining the incidence of transcription factor and *ABCC4* promoter interactions, with *ABCC4* promoter interactions proceeding through enhancers [90]. Surprisingly, however, in the absence of *Abcc4*, multiple genes involved in megakaryopoiesis (*Bcl2l1*, *Ccne2*, *Cd9*, *Il21r*, *Nfe2*) were found by RNAseq, to be upregulated compared to those in wild type mouse bone marrow megakaryocytes, suggesting that in vivo *Abcc4*^−/−^ megakaryocytes can compensate for the absence of *Abcc4*, resulting in near normal platelet levels in *Abcc4^−/−^* mice.

Lipid transport is important to the process of megakaryopoiesis, since both DMS formation and proplatelet extension involve significant reorganization of lipid membranes, and disruptions of lipid transport are therefore expected to affect platelet production. In effect, diet sourced extraneous fatty acids released by adipocytes may be taken up by platelets through CD36 and have been shown to sway platelet counts in vivo [91]. Some platelet membrane transporters do traffic lipids, with variable consequences for platelet numbers and size.

***ABCA1***. Tangier disease is a rare autosomal disease caused by mutations in *ABCA1*, a gene with more than 100 known mutations. Patients have a defect in mobilization of cholesterol efflux to high density lipoprotein (HDL), resulting in cholesteryl accumulation in body tissues and very low or virtually absent plasma HDL. Ubiquitin-mediated targeting for lysosomal degradation and phosphorylation appear to be two major modes of regulation of ABCA1 levels and activity. [92]. Addition of ubiquitin at Lys63 (K63), e.g., by Listerin E3 ubiquitin ligase 1, catalyzes K63-linked polyubiquitination at ABCA1 residues K1884/K1957, to counter ESCRT-mediated lysosomal degradation of ABCA1 induced by oxidized LDL (oxLDL), and instead stabilizes the protein [93]. In resting RAW264.7 cells, the COP9 signalosome may serve to protect ABCA1 from degradation under basal conditions [94]. Phosphoregulation of ABCA1 may be executed by protein kinase A (PKA), protein kinase C (PKC), Janus kinase 2 (JAK2) and casein kinase (CK2), with all these proteins known to be present in platelets. Most ABCA1 mutations reside in extracellular domains (ECDs) [8] and thus may escape normal post-translational management. Moreover, cell-specific processes may dominate, and it is currently uncertain which are the paramount mediators in megakaryocytes and platelets.

Tangier patients face multiple problems such as neurodegenerative disease, splenomegaly, and cardiovascular disease, and may also suffer from bleeding disorders and thrombocytopenia [95]. A role for ABCA1 in megakaryocyte and platelet integrity in Tangier disease was postulated. *ABCA1* mRNA was found to be upregulated during human megakaryocyte differentiation and was hypothesized to alter lipid raft associated receptor clustering [96] and the reorganization of cholesterol domains. Furthermore, quantitative electron microscopy studies have confirmed an irregular distribution, with ABCA1 microdomains on the cell surface being more prone to forming exocytotic vesicles [97]. Occasionally, patients present with a more limited subset of symptoms. Recently a novel *ABCA1* variant was detected in a Spanish family with HDL deficiency, mild to severe thrombocytopenia and no CVD. Primary cell cultures from homozygous patient carriers revealed impaired megakaryocyte differentiation associated with aberrant accumulation of neutral lipids in megakaryocytes, indicating that the thrombocytopenia was likely not due to platelet clearance [98].

***ABCG4***. Another membrane transporter mediating cholesterol efflux, ABCG4, has been linked to megakaryopoiesis, however, with an opposite outcome. Transfer of *Abcg4* deficient bone marrow into *Ldlr*^−/−^ mice resulted in thrombocytosis and increased atherosclerotic lesions. Although *Abcg4* mRNA was primarily detected in megakaryocyte–erythroid progenitors (MEPs), *Abcg4* deficiency rendered the mice more responsive to TPO in vivo, consistent with increased c-MPL receptor levels on megakaryocyte progenitors, supporting this pathway as the mechanism underlying increased platelet production and levels in *Abcg4*^−/−^ mice. [20].

***ABCB6****. ABCB6* expression is mostly limited to megakaryocyte–erythroid progenitors (MEPs). Similarly to *Abcg4* deficiency, transplant of bone marrow from *Abcb*^−/−^ mice into *Ldlr*^−/−^ recipient mice resulted in an enhanced MEP population, increased proliferation of megakaryocyte progenitors, higher platelet counts and increased mean platelet volume. Lack of *Abcb6* led to amplified reactive oxygen species generation and platelet–leukocyte interactions that provoked an increased incidence of atherosclerosis [13]. These studies highlight how disruptions in membrane transporters expressed at different stages of platelet production may be problematic for maintenance of stable plasma platelet populations, platelet reactivity and cardiovascular disease outcomes.

***ABCG5/ABCG8***. The half transporters ABCG5 (Sterolin-1), and ABCG8 (sterolin 2) form heterodimers that play essential roles in regulating cell membrane fluidity [24]. Mutations or deletions of these transporters are causative for Sitosterolemia, a rare autosomal recessive disease that can lead to macrothrombocytopenia, impaired excretion and thus increased retention of plant sterols and cholesterol [21,22]. *Abcg5* and/or *Abcg8* deficient mice serve as models for Sitosterolemia. In *Abcg8*^−/−^ mice fed a high sterol diet, the percentage of high ploidy megakaryocytes (ploidy > 128N) as well as immature megakaryocytes (ploidy 2N and 4N) was increased, with resultant macrothrombocytopenia. In tandem, slight decreases in expression of FILAMINA, GPIbα, both known to regulate platelet size and morphology, were observed. Unexpectedly, extramedullary hematopoiesis was also detected in the spleen, liver, and lungs of high sterol fed mice [25]. Deletion of the partner transporter in mice (*Abcg5*^−/−^) yielded a 68% reduction in platelet count amidst deterioration of the DMS [23] that was not fully corrected by transplantation of wild type bone marrow, thus emphasizing that plasma platelet clearance defects may also contribute to the macrothrombocytopenia. In patients, accompanying symptoms commonly include hypercholesterolemia, childhood-onset xanthomas, arthritis, and hemolytic anemia, as well as bleeding risk with platelets featuring macrothrombocytopenia [99]. However, atypical presentations of sitosterolemia may occur, as was diagnosed in a 6.5 year-old female patient during the evaluation of thrombocytopenia, where no accompanying symptoms were detected. Fortunately, dietary sterol restriction together with Ezetimibe, a drug that lowers LDL and interferes with cholesterol absorption from the intestine, has seen some success in improving hematologic response, with platelet counts increasing from 58,000/mm^3^ to 140,000/mm^3^ following therapy [100,101]. These cases illustrate the importance of early diagnosis, and the highly effective correction of platelet counts and related defects possible with appropriate treatment in Sitosterolemia.

***SLC35a1*.** SLC35a1 encodes the cytidine-5′-monophosphate (CMP)-sialic acid transporter that transports CMP-sialic acid from the cytoplasm into the Golgi apparatus for sialylation. The loss of SLC35a1 and subsequent elevated levels of hyposialylated glycoforms (3-sialo to 1-sialo), appears to yield a congenital disorder of glycosylation with impaired maturation of protein-linked N-glycans in the Golgi apparatus. Patients commonly exhibit macrothrombocytopenia and megakaryocytes with few demarcation membranes, and suffer from additional symptoms including neutropenia, opportunistic infections, and delayed psychomotor development [44,45,46]. Thrombocytopenia appears compounded by an additional accelerated clearance of unsialylated platelets by liver Kupfer cells [102]. Thus, slc35a1 may play a role in platelet generation and stability.

***SLC2a1/SLC2a3***. Not surprisingly, glucose metabolism and glycolysis can impact platelet numbers. GLUT1, and GLUT3 are the only Class I glucose transporters expressed in platelets [103]. A link between glucose metabolism in *Slc2a1*/*Slc2a3* double KO mice and stable platelet numbers is clear from the thrombocytopenia in the double KO animals-however the normal numbers in mice with the individual GLUT1 or GLUT3 KO mice indicate some redundancy. Although no changes in megakaryocyte number were observed in femurs or spleens of double KO mice, in vitro culture of BM differentiating to megakaryocytes indicated impaired proplatelet formation, while depletion of platelets resulted in delayed recovery in double KO mice compared to wild type mice. This discrepancy may be attributed to complete inhibition of glucose uptake, a shift to increased mitochondrial oxidative metabolism of non-glycolytic substrates, destabilization of calcium homeostasis, and activation of a calpain-mediated cell death and clearance pathway [26].

***SLC16a1/*MCT1**. When glucose is limited, e.g., during fasting, vigorous exercise, or a ketogenic diet, fat is broken down by the liver to produce beta-hydroxybutyrate (β-OHB or BHB), a primary ketone body, and may fuel increased megakaryopoiesis with production of normal size and morphology platelets. Genetic depletion of the megakaryocyte-specific ketone body transporter monocarboxylate transporter 1 (MCT1; SLC16a1) or its pharmacological targeting blocked β-OHB-induced thrombopoiesis in mice. This effect of MCT1 and β-OHB was tested in a ketogenic diet which enhanced histone H3 acetylation of promoters of platelet biogenesis genes, *GATA-1* and *NFE-2* in bone marrow megakaryocytes to induce thrombopoiesis. In contrast, inhibition of MCT1 or its depletion in mice blocked β-OHB-induced thrombopoiesis. For patients with chemotherapy induced thrombocytopenia, MCT-1 and dietary supplementation with β-OHB is thus proposed as a means to counter low platelet counts, particularly since no thrombocytosis is seen in healthy control volunteers [42].

***SLC11a2/*NRAMP2/DMT1**. Divalent Metal Transporter1(DMT1). **SLC11A2/DMT1**. The divalent metal transport 1 (DMT1) is a ferrous iron (Fe^2+^) transporter expressed in platelets, that may also transport Mn^2+^ and other divalent cations [40]. Upon acidification of the endosome, iron is released from transferrin and transported across the endosomal membrane into the cytosol by DMT1. DMT1 is serine phosphorylated basally in most cells. Site-directed mutation at N-terminal S^43^A in model cells overexpressing DMT-1 abolished basal phosphorylation and impeded Fe^2+^ uptake. The G-protein coupled receptor, CB2, known to be expressed in platelets [104] negatively regulates phosphorylation at this site, and disrupts iron transport, thereby potentially limiting oxidative stress under inflammatory conditions [105]. Proteasomal degradation also serves to regulate iron flux [41].

While the divalent metal transporter DMT1 is expressed as four different isoforms, it is the DMT1 isoform lacking the IRE response element (DMT1B-nonIRE) that is of relevance to hematopoiesis [106]. DMT1B-nonIRE was found to regulate normal hematopoiesis and leukemia through activation of the NOTCH-MYC pathway. Accordingly, severe bone marrow failure in *Dmt1*-nonIRE knockdown mice yielded a two-fold increase in the number of platelets due to suppression of NOTCH/MYC pathway activity, but hindered development across lymphoid and other myeloid lineages. Interestingly, this isoform of DMT1 has also been found in the outer mitochondrial membrane (OMM), where it can import metals into the mitochondria to play a role in oxidative metabolism [107]. In erythrocytes, loss of DMT1 results in increased glycolysis and increased oxidative stress [108]. Currently, it is unknown if it is also a factor in platelet generation defects in carriers of this isoform.

***SLC35d3***. SLC35d3 encodes an orphan nucleotide sugar transporter protein that in platelets directs contents to dense granules. Normally, membrane transporters may import contents from the megakaryocyte or platelet cytosol to dense granules, but these granules are either lacking content or absent in Hermansky–Pudlak syndrome (HPS). A spontaneous insertion of an IAP element within exon 1 of *Slc35d3* is responsible for the ash-Roswell mouse model of HPS [56], however in human patients with symptoms of HPS, e.g., bleeding and reduced stored serotonin, only 2 patients of 22 harbored a heterozygous mutation in Slc35d3 (Q317H). To note, the bleeding phenotype seen in patients is also reproduced in SLC35D3-null mice which display defective dense granule formation. During mouse megakaryocyte differentiation, SLC35d3 expression is upregulated. By tracing SLC35d3 expression through dense granule synthesis, SLC35D3 delivery to nascent dense granules from early endosomes was observed as megakaryocytes matured from fetal liver cells [57]. SLC35D3 is unusual among SLC35 family members in having a long C-terminal cytoplasmic extension with two potential YxxØ consensus endocytic sorting signals. The Y^311^EDL, located in the cytosolic domain of SLC35D3, was found to be important for its association with the AP3 sorting complex and its sorting into dense granule precursors [58], and disruption of this sequence may cause its aberrant localization.

***SLC22a1***. The hydroxyurea (HU) transporter family comprises SLC22a1, A2, A3 (OCT and OAT’s), and is important for the transmembrane movement of HU [109,110]. HU is a treatment sometimes applied to counter thrombocythemia in myelodysplastic syndromes or sickle cell anemia, as it acts on hematopoietic stem cells to suppress DNA synthesis and cell proliferation in the bone marrow, thereby diminishing the occurrence of blood clots. Although hydroxyurea transporters have not been clearly identified and validated in platelets, megakaryocyte–erythroid progenitor cells do express members of this subset of SLC transporters [109].

## 4. Transporter Roles in Platelet Activation and Function

*Platelet Activation.* A major reason that platelets can act so rapidly is that many of their functional molecules are stored in specialized granules that can be easily mobilized for fusion with the plasma membrane upon activation. Alpha granules are the most abundant platelet granules and contain proteins and other biomolecules involved in adhesion, coagulation, inflammation, vascular repair, and communication with other cells. Important alpha-granule cargo includes von Willebrand factor (VWF), fibrinogen, P-selectin, platelet factor 4, chemokines, anti-microbials and growth factors [111]. Dense granules are smaller but are especially important for amplifying platelet activation because they store ADP, ATP, calcium, serotonin, and polyphosphates [111]. When platelets are activated, these granules fuse with the platelet membrane and release their contents into the local environment [112]. Platelets also contain lysosomes, which carry enzymes involved in degradation and remodeling. In this way, platelets function not only as adhesive elements of a clot, but also as rapid secretory cells that intensify hemostatic and inflammatory signaling at the injury site.

Platelet activation begins when platelets encounter subendothelial adhesion molecules that are normally hidden from circulating blood. At a site of vascular injury, exposed collagen and VWF provide the first adhesive cues, and fibrinogen deposited from plasma may be recognized by unactivated αIIbβ3 to initiate outside-in signaling and platelet spreading [113]. The GPIb-IX-V receptor complex binds VWF and helps platelets tether to the injured vessel wall, especially under high shear conditions [114,115,116]. Collagen receptors such as GPVI and integrin α2β1 then strengthen adhesion and promote intracellular signaling [117,118]. In parallel, soluble agonists amplify activation: ADP released from dense granules signals through platelet P2Y1 and P2Y12 receptors [119], thrombin activates protease-activated receptors (PAR) [120], and thromboxane A2 acts through the thromboxane receptor, TP [121], with signaling by these receptors mediated through G-protein coupled receptors (GPCR) [117]. Although these pathways begin with different stimuli, they converge on a shared functional outcome, i.e., activation of the key integrin αIIbβ3 [113]. Once activated, αIIbβ3 binds fibrinogen and von Willebrand factor on flowing platelets, forming bridges between neighboring platelets and recruiting them into growing platelet aggregates [117,122,123,124].

Ion flux is an important part of platelet activation, with Ca^2+^ serving as an important intracellular signal. After stimulation by agonists such as collagen, thrombin, ADP, or thromboxane A_2_, platelets may release Ca^2+^ from intracellular stores and allow additional Ca^2+^ entry through store-operated channels such as the STIM1–Orai1 pathway [125,126]. This rise in cytosolic Ca^2+^ activates key signaling pathways to support shape change, granule secretion, thromboxane generation, integrin αIIbβ3 activation, and procoagulant membrane changes [52]. Other ions also support platelet function: K^+^ and Cl^−^ channels regulate membrane potential and platelet volume [127,128], Na^+^/H^+^ exchange contributes to intracellular pH regulation [129], Mg^2+^ can modulate platelet aggregation and adhesion [130,131], while Zn^2+^ is stored in platelet granules and can be released during activation to influence aggregation and platelet-dependent coagulation responses [132,133].

**Figure 2:**
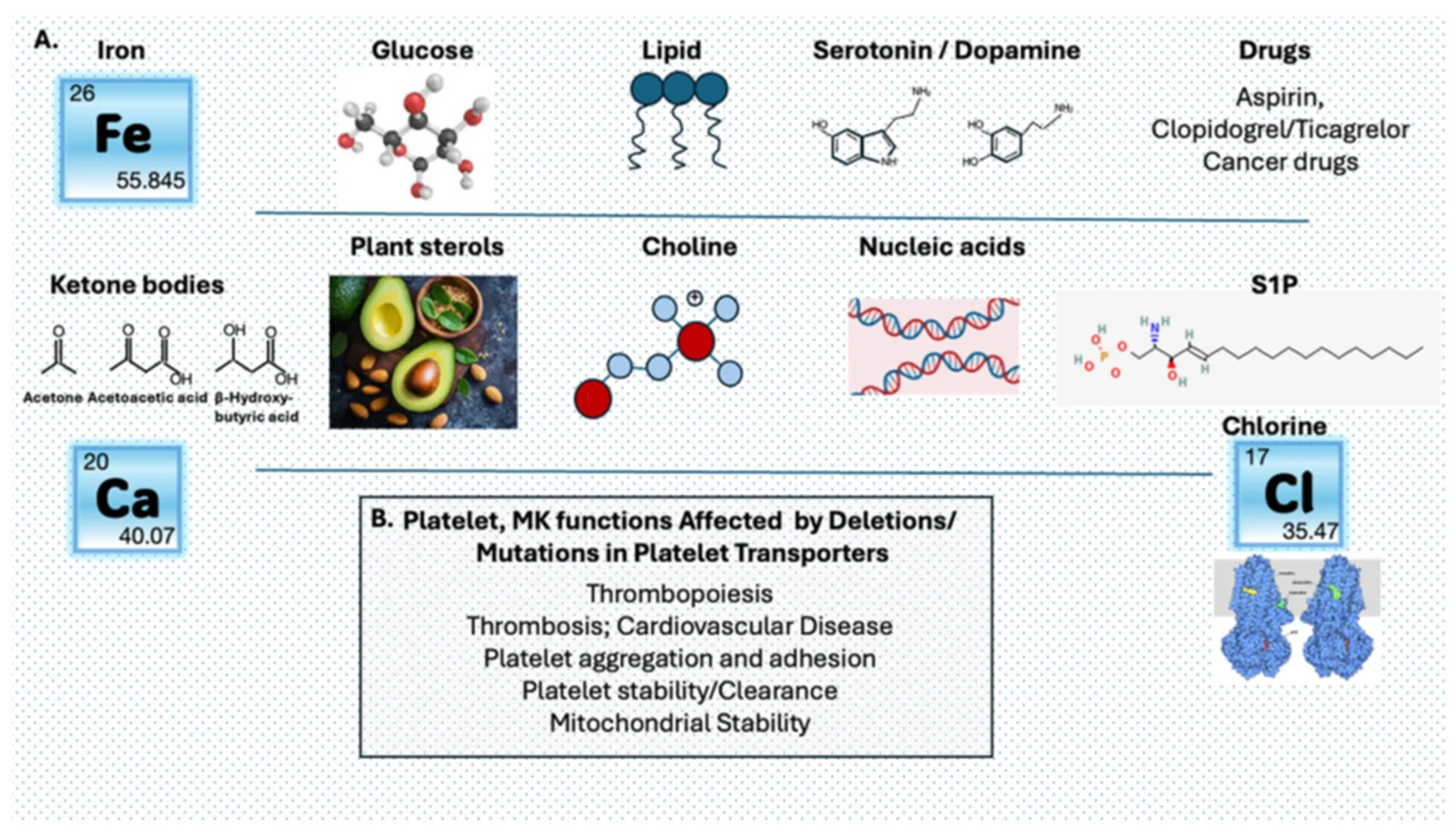
Relevant platelet substrates transported and discussed. Platelet transporters traffic a very wide array of substances, as illustrated (**A**), with impacts on platelet and megakaryocyte (MK) production and function (**B**).

Genetic mutations or deletions of membrane transporters may manifest as multi-symptom diseases (Figure 1D) that display perturbed canonical platelet functions as well as disruption of normal platelet interactions with other cell types, particularly immune cells. Figure 2 provides a summary of membrane transporter substrates and their impact on platelet development and function.

***ABCC4/*MRP4**. ABCC4 has been shown to play a role in cyclic nucleotide and prostaglandin export and is involved in clearance of antithrombotic drugs (see Section 6). It was also postulated to orchestrate the release from dense granules to plasma of sphingosine-1-phosphate (S1P), a concentration-dependent modulator of platelet activation; at low concentrations it may amplify, and at high concentrations it may inhibit platelet responses [15,16]. Indeed, a correlation between increased MRP4 and plasma S1P was described in convalescent patients post Covid19 [134]. In certain disease states including HIV MRP4 may be upregulated and MRP4 inhibition can directly reduce platelet activity and diminish the release of inflammatory mediators, including S1P [135]. As a consequence, the MRP4 transporter has emerged as a potential target to curb undue platelet activation. Several inhibitors have been developed that interfere with the action of MRP4 and affect varying modulators of platelet activation. The MRP4 inhibitor MK571 elevated intracellular concentrations of cAMP and cGMP and potentiated their repressive effects on platelet activation [136] driven by forskolin and sodium nitroprusside (agents that increase cAMP and cGMP, respectively), that was reversible by addition of a PKA inhibitor [137]. A different MRP4 inhibitor, Ceefourin-1, also increased cytosolic cAMP and cGMP levels, reduced calcium influx, inhibited ADP- and collagen-induced platelet aggregation by 30–50%, and reduced thrombus formation in a flow chamber assay [138], supporting the results of chemical targeting of MRP on platelet activity. *Abcc4^−/−^* knockout mice, reproduced the anti-platelet effects seen with inhibitors, displaying decreased platelet adhesion to collagen in shear flow and reduced thrombus formation in vivo in a ferric chloride model, potentially driven by reduced GPVI membrane localization [139]. Mechanistically, the PKA catalytic subunit was found to associate with MRP4. Upon PMA activation the catalytic subunit was liberated from MRP4 and the PKA regulatory subunit, and ABCC4 inhibition elevates cAMP and increase PKA activity [89]. Furthermore, MRP4 forms extensive protein networks through its PDZ domain, that are relevant for MRP4 localization and cAMP transport efficacy [140].

***ABCG5/ABCG8***. Premature cardiovascular disease (CVD) is a common feature in Sitosterolemia patients, with heterozygotes also exhibiting increased risks for CVD due to high plasma sitosterol and LDL-C levels. In the spontaneous mouse model of Sitosterolemia, thrombocytopenia and cardiovascular disease “*trac*”, mouse platelets had accumulation of free sterols in platelet plasma membranes that rendered them hyper-reactive but with unanticipated increased bleeding time, reduced adhesion, and thrombus formation [141]. Although surprising at first, *trac* mouse platelets also exhibited increased fibrinogen binding and loss of integrin receptor αIIβ3 availability through constitutive internalization in addition to enhanced GPIbα shedding leading to impaired adhesion to vWF and inability to form stable thrombi [25].

***ABCC7*/CFTR**. Cystic fibrosis (CF) is a severely debilitating disease affecting 100,000 people globally, that historically was associated with high mortality in young adulthood. It is characterized by acute lung inflammation that can lead to terminal respiratory failure, and is caused by mutations in the *ABCC7* gene, more commonly referred to as the cystic fibrosis transmembrane conductance gene (CFTR). Although classified as an ABC transporter, it is unusual in that it functions as a chloride channel that regulates ion and water secretion and absorption in epithelial tissues.

The regulatory domain of CFTR harbors protein kinase phosphorylation sites utilized by PKA, and SYK [142] and CFTR activity may also be controlled by endocytosis/recycling and degradation. The F508del mutation in the NBD1 domain, shared by >90% of cystic fibrosis patients, results in misfolded CFTR at the cell surface that was thought to be cleared via ubiquitin-mediated lysosomal targeting [143]. However, more recent work suggests that almost complete ER-associated degradation (ERAD) occurs through recognition of misfolded, mutated CFTR by a heat shock Hsp27 protein and conjugation of the small ubiquitin-like SUMO, that is then targeted by ubiquitin E3 ligase, RNF4 (RING finger protein 4) and directed to the proteasome [144]. Model cells have been used to probe post-translational modifications of CFTR and most regulatory molecules described or their homologues are expressed in platelets, thus pathways are expected be relevant in platelets.

While it was known that CF patient platelets displayed hyperaggregability and some thrombocytosis [18], a closer link between the disease and platelets was established following the identification of the transporter in human platelets [19], and generation of a platelet-specific CFTR knockout mouse. In the study by Ortiz-Munoz et al., the recently recognized role for platelets in inflammation led the authors to hypothesize that CFTR mutated platelets might be provoking the severe inflammation observed in patients. Indeed, the results indicated an exciting new role for platelets in the exacerbation of acute lung inflammation following airway challenge in a CFTR platelet-specific KO. This could be traced to increased calcium entry, agonist-induced hyperactivation, neutrophil activation and NET formation. Furthermore, treatment of human platelets with the cell surface CFTR chloride channel inhibitor, Ivacaftor, was able to partially reduce platelet activation, as reported by markers CD62P and CD63 [145]. In a later study of coronary artery disease (CAD) patients, an additional role for platelet CFTR in CAD was postulated, since patient platelets had a significant decrease in CFTR levels and increased intracellular chloride levels. Platelet-specific *Cftr^−/−^* platelets with increased chloride ion concentrations also exhibited accelerated thrombus formation in FeCl3-induced injury, increased platelet aggregation and ATP release. Surprisingly, in these mice, there was significant upregulation of P2Y12 and PAR4 receptors, both critical for in vivo thrombus formation, that was attributed to serum and glucocorticoid-regulated kinase (SGK1)-mediated NF-κB transcriptional activation in megakaryocytes [146]. A greater understanding of molecular characteristics and sources of CFTR abnormalities will aid in developing gene editing of specific mutations in relevant cells, to further extend lifespan in CFTR patients.

***SLC6A4/*SERT**. Serotonin modulates function in several cell and organ systems, including platelets, brain and liver, and SERT has been found to be expressed along with the dopamine transporter (DAT) in megakaryocytes derived from Meg-01 cells [35]. In the periphery, serotonin (5-HT) is produced by enterochromaffin cells in the gastrointestinal tract, released into the plasma, and quickly taken up by platelets to prevent excesses in the circulation. Serotonin is taken up via the plasma membrane serotonin transporter (SERT) and stored in dense granules. The transporter has been found to undergo both serine/threonine, and tyrosine phosphorylation, and the effective kinases reported in other cell types are mostly also present in platelets. Tyrosine phosphorylation at Y47 and Y142 is fundamental to both SERT protein stability, and serotonin transport function, and has been proposed to be mediated by SRC family kinases, known regulators of platelet adhesion [36]. Serine phosphorylation by p38 MAPK has also been shown to promote serotonin uptake [147], and Thr-276 within a transmembrane helix has been identified as a stimulated cGMP phosphorylation site that must be made conformationally accessible in the transport cycle, to enhance transport [148,149].

Functional polymorphisms in the *SLC6A4* gene may yield higher or lower than normal serotonin levels. An association between the SERT polymorphism containing a long promoter variant that causes high SERT expression (LALA), and lower risk of ischemic stroke/TIA was found in a large cohort of 834 patients [37]. This diverges from the previously described risk of myocardial infarction associated with SERT polymorphisms [150]. However, cardiovascular and cerebrovascular diseases have different etiological profiles. In patients chronically taking selective serotonin reuptake inhibitors (SSRIs), depletion of platelet serotonin reduces 5-HT’s amplification of platelet activation resulting in lower platelet aggregation and a mild bleeding phenotype. In *Slc6a4^−/−^/*SERT^−/−^ mice, reduced blood levels of serotonin similarly led to slightly prolonged bleeding times, and whereas stenosis induction in the vena cava induced large thrombi in wild type mice, thrombus size was significantly reduced in the *Slc6a4^−/−^/*SERT^−/−^ mice [38]. These mice were particularly insensitive to activation with lower doses of ADP, as expected, given the lack of synergistic potentiation by 5-HT, and reduced activation of G-proteins coupled to 5-HT2ARs [151].

***SLC58a1*/MAGT1.** MAGT1 is causative for an X-linked immunodeficiency (XMEN) disease, a rare genetic immunodeficiency affecting males, caused by loss of function mutations in the magnesium transporter, MAGT1, leading to an inborn error of glycosylation with symptoms including hemorrhagia, susceptibility to Epstein–Barr virus, intellectual deficiencies, and neoplasia. MAGT1 serves as an accessory subunit for an oligosaccharyltransferase complex in the ER [49,152]. Much of what is known of platelet dysfunction in this disease has been gained from studies of patients with XMEN disease, with some variability in patient symptoms noted. Kauskot et al. [153] described two young male patients with impaired platelet glycosylation, severe platelet morphological and functional abnormalities (hemorrhagic), reduced platelet aggregation, integrin αIIbβ3 activation and PKC-dependent serine phosphorylation in response to stimulation with canonical platelet agonists at all concentrations. Decreased molecular weight of glycoproteins GPIbα, αIIb, and GPVI were observed, likely due to decreased glycosylation of these receptors. More recently, allogeneic stem cell transplants are being tested as a cure for XMEN disease but have encountered high mortality, with some patients suffering from intracranial hemorrhage or posterior epistaxis with hemorrhagic shock early post-transplant. Successful transplants appeared to be aided by maintaining platelet counts above 30 × 10^9^/L during transplant [154].

***SLC59a2*/MFSD2B**. The major facilitator superfamily orphan transporter 2b (MFSD2B), a cation dependent lipid transporter, is also implicated in the export of S1P at the plasma membrane from red blood cells and platelets and parallels the ABCC4 pathway that supports secretion of S1P from dense granules. Signaling by S1P, a bioactive lipid, is relevant for platelet function [155]. Interestingly, unlike in *Abcc4*^−/−^ mice, in the platelet-specific *Mfsd2b*^−/−^ mouse, loss of MFSD2B provoked greatly elevated S1P accumulation in erythrocytes and platelets while reducing plasma S1P levels by 50%, and substantially decreasing thrombus formation [50]. A later study by Polzin et al. hypothesized that MFSD2B may dynamically regulate S1P levels during acute myocardial infarction (AMI), and that platelets may provide the burst of S1P during AMI that determines the extent of myocardial damage. Consistently, supernatant from activated platelets reduced infarct size in wild type mice after AMI in vivo; however, supernatant from activated *Mfsd2b^−/−^* platelets resulted in a 40% increase in infarct size, indicating a normally cardioprotective role for MFSD2B through modulation of S1P transport [51].

***SLC5a1/*, *SLC5a2/*SGLT1 and SGLT2**. A study by Wichaiyo et al. [33] investigated the roles of two sodium glucose cotransporter 2 (SGLT2) inhibitors on platelet functions, empagliflozin and dapagliflozin, commonly used in the treatment of T.2 diabetes and heart failure. Despite a lack of platelet expression of SGLT1 and SGLT2—they operate mainly in the kidney—these inhibitors exert anti-platelet effects by reducing collagen-induced platelet aggregation and lowering thrombin generation, indicating an indirect mode of action. Molecular docking suggested binding of both SGLT2 inhibitors to the catalytic subunit of PKA, yet only empagliflozin directly enhanced PKA catalytic activity. However, Dapagliflozin promoted increased eNOS expression, which may increase NO production, PKA activity and VASP phosphorylation. A further effect of dapagliflozin and another inhibitor, sotagliflozin, was shown in sickle cell disease mice, where long term administration of the drugs reduced vaso-occlusive events, in part due to lower platelet-neutrophil interactions [34]. Thus, non-platelet membrane transporters nevertheless may influence platelet activity, but further studies are needed to better elucidate exact mechanisms responsible.

***SLC2a1/*GLUT1*/SLC2a3/*GLUT3**. Platelet activation drives a significant increase in glucose uptake, glycolysis, and lactate production [156] suggesting that both oxidative phosphorylation and glycolysis may contribute to ATP production, the metabolic energy source that fuels platelet function. Of the two GLUT platelet receptors GLUT1 is primarily found at the plasma membrane. GLUT3 has higher expression than GLUT1 and is distributed primarily to alpha granules (85%) in unstimulated platelets, but translocates to the plasma membrane upon platelet activation [30,31]. Initial trials to decipher the role of these two glucose transporters were focused on GLUT3 using a platelet-specific *Glut3*^−/−^ (ps*Glut3*^−/−^) mouse. Whereas glucose uptake in platelets isolated from these mice was only mildly reduced at baseline, thrombin-mediated glucose uptake was abolished. In fact, the psGlut3^−/−^ mice turned out to be protected in a collagen epinephrine pulmonary embolism survival model despite normal platelet numbers, and tail bleeding times. However, decreased α-granule release, clot retraction and spreading on fibrinogen together may have shielded the mice [32]. Surprisingly, ps*Glut1^−/−^* platelets revealed no changes in basal or thrombin-mediated glucose uptake relative to controls, and there are patients with GLUT1 Deficiency syndrome who suffer from brain energy deficiency symptoms without hematological abnormalities [27]. Thus, unique and overlapping roles may exist for GLUT1 and GLUT3. Genetic depletion of GLUT1 in megakaryocytes may interfere with a c-MYC-dependent upregulation of glucose-regulated protein 75 (GRP75), a mitochondrial molecular chaperone that in platelets can trigger platelet activation and aggregation, indirectly priming platelets to a pro-metastatic phenotype with increased adherence to tumor cells [157].

A more severe metabolic phenotype was found in the platelet-specific double GLUT1 and GLUT3 KO (ps*Glut1^−/−^*, *Glut3^−/−^)* mice, where glucose uptake was completely abolished and DKO platelets did not undergo glycolysis. Although in the absence of glucose uptake, alternative metabolic pathways may sustain cellular production of ATP, the energy currency, and maintain cellular viability, upregulation of these pathways through addition of alternate mitochondrial substrates was not able to rescue the decreased αIIbβ3 activation seen even at higher agonist concentrations, or the reduced α-granule number and release. ps*Glut1^−/−^/Glut3^−/−^* mice overall showed a more severe phenotype than ps*Glut3*^−/−^ mice and had significantly prolonged tail bleeding times, and reduced thrombus growth in a Fe3Cl3 model of arterial thrombosis but were similarly able to increase survival in a collagen–epinephrine model of pulmonary embolism. Thrombopoiesis was also impaired (see Section 4). Investigation of mitochondrial changes in freshly isolated ps*Glut1^−/−^*,*Glut3^−/−^)* mouse platelets indicated signs of metabolic stress: increased phosphorylated AMP-activated protein kinase (AMPK) and acetyl-CoA carboxylase (ACC), increased membrane potential and produced three-fold higher oxygen consumption rates in the presence of glucose, glutamate, and pyruvate. [26]. Glucose transport by GLUT1 when phosphorylated at serine 226 by PKA, or N-glycosylated may lead to rapid glucose uptake and agonist-induced relocalization to the plasma membrane in endothelial cells, red blood cells and model cells [28,29,158]. In GLUT1 deficiency syndrome, serine 226 is mutated and leads to a brain deficiency syndrome [27]. Whether and by which specific mechanisms these mitochondrial transformations contributed to the platelet functional phenotypes observed will need to be further elucidated.

**SLC8a (SLC24a)/NCX Sodium Calcium (Na(+)/Ca(2+))exchanger**. NCX is a bidirectional transporter that helps maintain stable cytosolic calcium levels by effluxing excess calcium from resting platelets and by raising cytosolic calcium upon stimulation with select agonists. Platelet cytosolic calcium levels are highly regulated and of great importance in controlling many aspects of platelet activation [52]. In MEG01 cells, transcription of NCX1, NCKX1, NCKX2, and NCKX5 and accompanying Na^+^/Ca^2+^ exchanger activity can be induced by TGFβ1 [54] through activation of p38 kinase, SGK1 and NFκB, although other family members, NCX1.3, NCX3.2 and NCX3.4, may also be expressed in platelets. Fibrinogen binding to integrin αIIbβ3 promotes NCX1 relocalization to the plasma membrane, association with the integrin and an NHE1-mediated local increase of sodium concentration that redirects NCX1 to pump out sodium in exchange for calcium influx [39]. During collagen activation, NCX1 and NCX3 but not NCKX1 were also found to transiently reverse transporter direction to promote Ca^2+^ influx [159]. In a separate study, NCX3 was conjectured to be the NCX that was primarily expressed in platelets and enabled PMA induced dense granule secretion and Ca^2+^ entry [53]. Thus, while some discrepancies in the specific NCX isoforms expressed or their actions have been reported, in part likely due to variations in experimental approaches and reagents, altogether, the studies support a role for select agonist-driven NCX transporter mediated Ca^2+^ entry.

**Mitochondrial Transporters as Metabolic Gatekeepers in Platelets**

In resting and normally activated platelets, mitochondria support platelet physiology through energy generation, calcium handling, redox signaling, and survival-related pathways. These mitochondrial functions help sustain energy-demanding platelet responses, particularly granule secretion and thrombus growth, while mitochondrial Ca^2+^ uptake and ROS generation regulate procoagulant conversion under stronger stimulation conditions [160,161,162,163]. Megakaryocytes are metabolically hyperactive and are particularly reliant on mitochondrial ATP generation and ROS to initiate proplatelet formation [164]. Thus, platelet mitochondria should not be viewed only as passive powerhouses; rather, they act as dynamic regulators that connect platelet metabolism with activation responses and thrombotic potential [160].

Platelet mitochondria require regulated transport of metabolites across mitochondrial membranes to maintain bioenergetic activity during resting and activated states. Because platelet activation is an energy-demanding process, mitochondrial transporters can influence platelet function by controlling substrate availability, oxidative phosphorylation, ATP generation, and downstream purinergic signaling [165]. Compared with mitochondrial channels and pores, platelet-specific mitochondrial transporters are less extensively characterized; however, emerging studies indicate that transporter-mediated metabolite flux can directly shape platelet activation, hemostasis, and thrombosis. In healthy megakaryocytes, mitochondria may even be exported and transferred via connexin 43 (CX43) gap junctions to BM mesenchymal cells to reduce glycolysis and platelet activity. In an unexpected connection to disease, this process is reduced in patients with sickle cell disease (SCD) and in SCD mice, further supporting the importance of mitochondria for normal platelet and megakaryocyte function [166].

***SLC44a2/*CTL2**. SLC44a2 is expressed in human and mouse platelets and localizes to the platelet mitochondrial fraction, where it supports choline transport into mitochondria. It was originally identified by GWAS studies as a risk factor for venous thromboembolism (VTE) [47]. Initial studies using *Slc44a2^−/−^* mice confirmed a role for the protein in thrombus formation in the cremaster muscle arteriole, as well as in the initiation of VTE in a neutrophil and VWF mediated stenosis model [167,168]. It is only later that the defects were traced to mitochondrial choline transport defects. *Slc44a2*-deficient platelets showed reduced mitochondrial respiration, ATP production, and ATP release upon platelet activation, together with impaired thrombin-induced aggregation, granule secretion, and integrin activation. These defects were associated with prolonged bleeding and reduced arterial and venous thrombosis. The recovery of platelet activation following exogenous ADP supplementation suggested that SLC44a2 primarily supports platelet responses by maintaining mitochondrial ATP synthesis and purinergic signaling [48].

***MCU*.** The mitochondrial calcium uniporter (MCU), located in the inner mitochondrial membrane, mediates Ca^2+^ uptake into the mitochondrial matrix following platelet stimulation. Studies using platelet-specific MCU deficient mice have shown that mitochondrial Ca^2+^ uptake supports platelet activation, particularly downstream of GPVI and CLEC-2, in part through effects on mitochondrial ROS generation and signaling [169]. MCU also helps couple Ca^2+^ signaling to mitochondrial respiration and ATP production during platelet activation [170]. Under stronger stimulation, sustained mitochondrial Ca^2+^ uptake promotes mitochondrial permeability pore transition, membrane depolarization, and phosphatidylserine exposure, contributing to procoagulant platelet formation [164,171]. MCU therefore provides a mechanistic link between mitochondrial Ca^2+^ uptake, platelet activation, and procoagulant platelet formation.

***SLC8b1/NCLX*** (a mitochondrial sodium calcium exchanger). NCLX acts to pump Ca^2+^ out of mitochondria and balances Ca^2+^ influx through the mitochondrial Ca^2+^ uniporter (MCU) and any other influx pathways. Free Ca^2+^ concentration within the mitochondrial matrix ([Ca^2+^]_m_) is important for many cellular processes, including the rate of ATP production. Stimulation through ADP may induce Ca^2+^ influx into the cytosol, but additionally into the mitochondrial matrix through MCU. Although NCLX mRNA was present but low in human platelets, surprisingly, inhibition by the NCLX pharmacological blocker CGP37157 (CGP) nevertheless enhanced the ADP-induced platelet aggregation response, platelet degranulation and integrin αIIbβ3 activation [55].

## 5. Drug Resistance

Anti-platelet drugs such as aspirin and P2Y12 receptor inhibitors, clopidogrel and ticagrelor, are widely prescribed both as therapeutic and prophylactic agents to target undue platelet aggregation and thrombus formation. Clopidogrel is a prodrug that must be metabolized to its non-reversible active anti-P2Y12 antagonist form principally by cytochrome P450 enzymes [172], whereas ticagrelor is a direct-acting, reversible P2Y12 inhibitor. Reduced absorption or excessive excretion by non-platelet, nucleated cells can change the disposition and metabolic efficacy profile of the drugs against platelet activity. Membrane transporters have been implicated in regulating anti-platelet drugs as well as other anti-cancer and immunosuppressive therapeutics and have thus become drug targets in their own right.

***ABCB1/*Multidrug Resistance-1(MDR1)/P-glycoprotein (P-gp**). P-gp, is a transmembrane efflux drug transporter 1, that transports toxins, drugs and lipids. When overexpressed it is a significant contributor to the MDR phenotype [10], since several transport-active binding sites allow for broad substrate interactions. Its expression has been noted in some megakaryoblastic cell lines [11,12] and is surmised to be present at very low baseline levels in platelets. Nevertheless, its strong expression in the intestinal tract and elsewhere can control drug excretion, and thereby have a large impact on outcomes in patients treated with anti-platelet medications. The orally administered anti-platelet prodrug, clopidogrel, is rapidly absorbed into the intestine. However, the intestinal levels are further regulated by P-gp, which actively effluxes a portion of clopidogrel into the intestinal lumen, thus lowering its availability for diffusion across intestinal capillary beds for entry into the bloodstream and delivery to the liver for metabolism to its active form. Specific polymorphisms in *ABCB1* such as C3435T and C1236T largely produced by splice variations, alter cellular levels of P-gp, leading to increased intestinal retention of clopidogrel [173], and decreased drug activity. Thus, the drug’s reduced protective effect can raise the risks of cerebrovascular events, with a higher prevalence of clopidogrel resistance particularly noted in females versus males (62.5% vs. 37.5%) [174,175]. The presence of gain or loss of function CYP2C19 polymorphisms and the *ABCB1*/P-gp C3435 C > T polymorphism that reduces intestinal drug efflux were assayed in a cohort of 100 elderly patients with acute coronary disease (ACD) on dual aspirin and clopidogrel therapy. Patients harboring gain-of-function CYP2C19 mutations, in conjunction with absent or a single copy of the ABCB1 3435 C > T mutation, experienced reduced cardiovascular risk but increased bleeding, in contrast to patients with a loss of function CYP2C19 mutation and homozygous ABCB1 TT mutations, who suffered increased thrombotic events. Thus, joint monitoring of these polymorphisms in the ACD population should help direct anti-platelet therapy to improve mitigation of ischemic versus hemorrhagic risk [176].

Treatment with ticagrelor has been associated with low platelet aggregation and bleeding, along with dyspnea in conjunction with increased plasma creatinine concentration and impaired renal metabolism, producing fluid buildup in the lungs in carriers of the *ABCB1* T allele [177]. These adverse effects often discourage patient usage and require shifting to alternate anti-platelet therapy. In a reverse condition, an elderly patient heterozygous carrier of Abcb1 variant alleles, suffered a subarachnoid hemorrhage due to a six-fold reduction in P-gP transport rates and high plasma levels of dabigatran, a thrombin inhibitor that interferes with platelet activation [178].

***ABCC4/*Multidrug resistance protein 4 (MRP4)**. Aspirin inhibits cyclooxygenase-1 (COX-1), which catalyzes the conversion of arachidonic acid to prostaglandin H, a precursor of thromboxanes [179] and is a staple of cardiovascular protection for patients at risk. Unlike ABCB1, ABCC4 is highly expressed in platelets and overexpressed in patients with CVD. It mediates ATP-driven transmembrane extrusion of drugs and other compounds, including eicosanoids such as thromboxanes. Mutations in *ABCC4* affecting platelet efflux of aspirin can have serious consequences for CVD management. In a cohort of coronary bypass grafting surgery patients with reduced sensitivity to aspirin, those who presented with genetically mutated *ABCC4* had increased export of aspirin from platelets, thereby reducing its inhibition of COX-1 and its anti-platelet efficacy [180]. Additional mechanistic insights were gained into the role of MRP4 in thromboinflammation, where circulating S100A8/A9 induced upregulation of MRP4 through altered microRNA regulation, which accelerated the extrusion of aspirin, thus reducing its effective half-life [181]. Low dose aspirin can also trigger *ABCC4* gene expression by enhancing PPARα activity, leading to higher *ABCC4* gene transcription [182,183], thereby creating a feedback loop.

***ABCC1/*MRP1**. This transporter is expressed in platelets, but an export function described for anticancer drugs has not yet been observed in platelets [184].

## 6. Conclusions

Membrane transporters have wide impacts on cellular metabolism as they are responsible for cellular homeostasis of a wide range of metabolites, drugs and proteins. Genetic abnormalities in transporter genes may lead to multi-symptom diseases, e.g., Tangier Disease, XMEN, and Sitosterolemia that involve disruptions in platelet production and function. A recent highly impactful observation that platelet disruptions in *ABCC7*/CFTR can lead to exacerbated lung inflammation in cystic fibrosis patients, may prove to be a tool for monitoring treatment for a severe and previously fatal disease. Therefore, it is hoped that new biochemical, animal model and genomics studies will drive more research into this often-ignored group of platelet-relevant proteins in order to develop a clearer understanding of transmembrane protein interactions within the wider metabolomic landscape and to extend new avenues of treatment for patients.

## Figures and Tables

**Table 1:** Platelet-relevant ABC and SLC family membrane transporters.

Gene Name/ NCBI Gene ID	Alternate Name(s)	Substrate(s)	References	Associated Disease(s)
*ABCA1/19*	CERP	cholesterol	[7,8]	Tangier
*ABCA7/10347*	AD9; ABCX; ABCA-SSN	lipid transport	[9]	Alzheimer’s
*ABCB1/5243*	P-gp/MDR1	toxins, drugs and lipids	[10,11,12]	CVD
*ABCB6/10058*	MRP6	lipids; porphyrin	[13]	familial pseudohyperkalemia atherosclerosis
*ABCC4/10257*	MRP4	cyclic nucleotides	[7,14,15,16]	CVD
*ABCC7/820498*	CFTR	chloride	[17,18,19]	Cystic fibrosis
*ABCG4/64137*	WHITE2	cholesterol efflux	[20]	atherosclerosis
*ABCG5/64240*	sterolin-1	plant sterols	[5,21,22,23]	sitosterolemia
*ABCG8/6241*	sterolin-2	plant sterols	[24,25]	CVD
*ABCB10/23456*	EST20237, M-ABC2, MTABC2	unknown	[5]	
*SLC2A1/6513*	GLUT1	glucose	[26,27,28,29]	GLUT1 deficiency syndrome
*SLC2A3/6515*	GLUT3	glucose	[26,30,31,32]	
*SLC1A2/6506*	EAAT2	glutamate	[6]	
*SLC5A2/6524*	SGLT2	sodium-glucose	[33,34]	
*SLC6A3/6531*	DAT	dopamine	[35]	
*SLC6A4/6532*	SERT	serotonin	[36,37,38]	
*SLC9A1/6548*	NHE1	sodium/proton	[39]	
*SLC11A2/4891*	DMT1	iron	[40,41]	
*SLC16A1/6566*	MCT1	ketone bodies	[42]	
*SLC18A2/6571*	VMAT2	vesicular monoamine transporter	[6]	psychotic disease
*SLC23A2/9962*	SVCT2	ascorbic acid	[6]	
*SLC25A6/293*	ANT3	ADP/ATP	[43] (human only)	
*SLC35A1/10559*	CST	CMP-sialic acid transporter	[44,45,46]	
*SLC44A2/57153*	CTL2	choline transport	[47,48]	
*SLC58A1/84061*	MAGT1	magnesium	[31,49]	XMEN disease
*SLC59A2/388931*	MFSD2B	lipid	[50,51]	
*SLC8 A1/6546; A2/6546; A3/6547*	NCX 1–3	Sodium-calcium exchanger	[52,53]	
*SLC24 A1/9187; A2/25769; A3/57419; A4/123041; A5/283652*	NCKX 1–5	potassium-dependent sodium/calcium exchanger	[54]	
*SLC8b/80024*	NCLX	mitochondrial sodium calcium exchanger	[55]	
*SLC35D3/340146*	n/a	Nucleotide sugar	[56,57,58]	
*SLC6A12/6539*	BGT-1; GAT2	GABA and monocarboxylic acid	[6]	
*SLC1A1/6505*	EAAC1; EAAT3; DCBXA	Glutamate	[6]	

## Data Availability

No new data were created or analyzed in this study. Data sharing is not applicable to this article.
